# Assessing the Therapeutic Efficacy of Photodynamic Therapy, Fractional CO_2_ Laser and Its Combination in the Treatment of Onychomycosis

**DOI:** 10.1111/jocd.70417

**Published:** 2025-08-31

**Authors:** Hamed Abdou, Muhammad Mohsen, Mostafa Taha Eldestawy, Shady Mahmoud Attia Ibrahim

**Affiliations:** ^1^ Al‐Azhar Faculty of Medicine Cairo Egypt

**Keywords:** antifungal treatment, combination therapy, dermatology, fractional CO_2_ laser, nail infection, onychomycosis, photodynamic therapy

## Abstract

**Background:**

Onychomycosis, a common fungal nail infection, poses challenges for traditional treatments due to long durations, liver toxicity risks, resistance, and poor nail penetration. So, alternative management approaches, which could involve combination therapy or methods to enhance topical drug delivery for optimal treatment efficacy, should be considered.

**Aims:**

This study evaluates the effectiveness of combining photodynamic therapy (PDT) with fractional CO_2_ laser treatment.

**Patients/Methods:**

Forty‐five patients with onychomycosis were included in the study. Patients were randomly assigned to three groups: PDT alone, fractional CO_2_ laser alone, and a combination of both. The treatment efficacy was evaluated based on clinical and mycological examinations and patient satisfaction.

**Results:**

The combined therapy group showed the best results, with 86.7% testing negative for onychomycosis and 26.7% achieving full nail clearance. Posttreatment satisfaction levels showed no significant differences among groups (*p* = 0.198).

**Conclusion:**

The combination of PDT and fractional CO_2_ laser appears more effective than either therapy alone, though further research is needed to confirm long‐term benefits. Incorporating combined therapy into clinical practice could enhance treatment outcomes for patients with onychomycosis.

## 1 Introduction

Onychomycosis, a prevalent nail disorder globally, is identified as a chronic fungal infection of the nail caused by dermatophytes, non‐dermatophytes, or yeast pathogens [1]. Clinically, onychomycosis accounts for up to 50% of all nail pathologies and is marked by nail discoloration, thickening, and defects, leading to significant physical and psychological impacts [2].

Oral and topical antifungal treatments remain the primary options for managing onychomycosis; however, they have various drawbacks. Oral antifungal treatments require prolonged therapy and can cause liver toxicity or cardiac issues, despite their high cure rate. Conversely, topical antifungal treatments are less effective due to the difficulty of penetrating the nail plate [3].

Therefore, there is a need for alternative management approaches, which could involve combination therapy or methods to enhance topical drug delivery for optimal treatment efficacy [4]. Previous studies have indicated that combining fractional CO_2_ laser with topical medications is more effective for treating skin conditions such as vitiligo, warts, melasma, alopecia, and onychomycosis [5].

Photodynamic therapy (PDT) is a noninvasive treatment used for various diseases. It involves three components: a photosensitizer, light, and oxygen. The photosensitizer selectively accumulates in abnormal or infected tissues without harming healthy cells, making it increasingly used as an antimicrobial for superficial infections [6].

The potential advantages of PDT include avoiding the side effects associated with systemic therapy, its rapid action, minimal side effects, and its ability to be combined with other treatments. We hypothesized that combining fractional CO_2_‐assisted PDT could be a valuable addition to treat onychomycosis.

## 2 Patients and Method

From October 2022 to March 2024, forty‐five patients diagnosed with onychomycosis were enrolled at the dermatology clinics of Al‐Azhar University Hospitals. Exclusion criteria included patients under 18 who had used topical antifungals in the past month or systemic antifungals in the last 3 months. Patients with other nail conditions like nail psoriasis, eczema, or lichen planus, and those with immune system issues, including HIV, organ transplants requiring long‐term immunosuppressants, chemotherapy, radiotherapy, or methylene blue allergies, were excluded. All participants provided informed consent after understanding the study's purpose.

Patients were randomly assigned to three groups using a computer‐generated randomization sequence with equal allocation (1:1:1). Group A had biweekly sessions of conventional PDT with 2% methylene blue solution (Alpha Chemical Co. LTD), applied for 30 min under dim lighting without occlusion before irradiation (2 sessions/month), using a red‐light source (Lasotronic/660 nm, 50 mW), applied for 10 min per affected nail, which were pretreated with urea 40% 24 h before PDT. Group B received fractional CO_2_ laser treatment (10 600 nm, Smart Xide, Punto, DEKA, Florence, Italy) with a power of 10 watts, pulse duration of 500 μs, spacing of 700 μm, and Stack 3 for four sessions at 3‐week intervals; each session included two passes of the laser over the affected nail. It should be noted that laser treatment was applied only to the nail plate surface, with no direct application made to the subungual tissue. Group C received a combination of fractional CO_2_ laser followed by conventional PDT with 2% methylene blue in the same session every 3 weeks.

The treatment efficacy was evaluated based on clinical and mycological examinations and patient satisfaction. Photographs were taken using a digital camera (Nikon D5300, Japan) under consistent settings, lighting, and nail positions at baseline and 12 weeks after starting therapy. Clinical assessment was performed using the Onychomycosis Severity Index, where mild nail involvement scored 5 or less, moderate involvement scored 6 to 15, and severe involvement scored 16 to 35. Treatment efficacy was determined by comparing infected area scores at baseline and 12 weeks (Table 1) [7].

**TABLE 1:** Onychomycosis severity index.

Area of involvement		Proximity of disease to matrix		Presence of dermatophytoma or subungual hyperkeratosis > 2 mm	
Affected nail, %	No. of points	Amount of involvement from distal edge	No. of points	Present	No. of points
0	0	< ¼	1	No	0
1–10	1	1/4–1/2	2	Yes	10
11–25	2	1/2–3/4	3		
26–50	3	> 3/4	4		
51–75	4	Matrix involvement	5		
76–100	5				

Mycological assessments were conducted at baseline and 12 weeks into therapy. Specimens, such as subungual debris, were collected from the affected nail areas and placed on clean glass slides. A drop of 20% potassium hydroxide (KOH) and 40% dimethyl sulfoxide (DMSO) was applied to enhance sensitivity and soften keratin without heat. A cover slip was placed on top with gentle pressure to remove excess solution. The samples were then examined under a microscope for fungal elements, including filamentous, septate, or aseptate hyphae, branched hyphae with or without arthrospores, and yeast cells.

Nail scrapings were also cultured on Sabouraud Dextrose Agar (SDA) with and without cycloheximide to identify pathogenic fungi and confirm infection. The cultures were incubated at 28°C and 35°C in a warm, humid environment and checked weekly for at least 4 weeks before being reported as positive or negative based on fungal growth.

A needle mount technique was used for detailed observation of fungal structures. A small portion of the colony was placed on a slide with a drop of lactophenol‐cotton blue (LCB), covered with a cover slip, and tapped to spread the hyphae. The sample was examined using lower (×10) and higher power (×40) objectives, with the oil immersion (×100) objective used for more detailed features.

**FIGURE 1:**
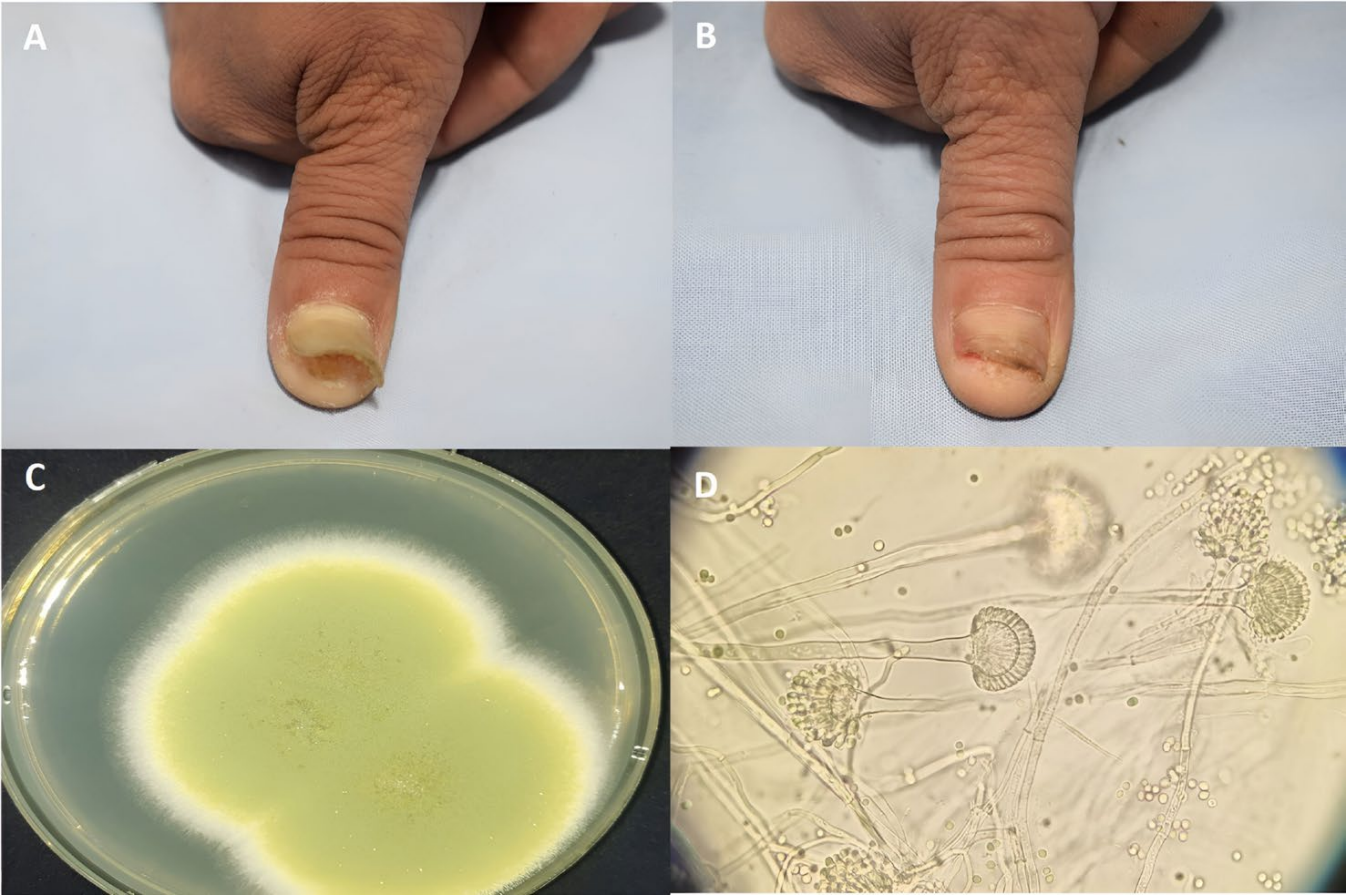
Patient with DLSO (A) Before treatment (B) After Photodynamic therapy only. (C) The gross morphology of Aspergillus flavus shows a large colony of velvety, downy, or powdery surfaces, with green to black colors. The color typically darkens with age. (D) Microscopic morphology of Aspergillus flavus shows slender conidiophores bearing small brown spherical vesicles with pigmented spore heads containing numerous large, globose biserite conidia on phialides and metulae.

**FIGURE 2:**
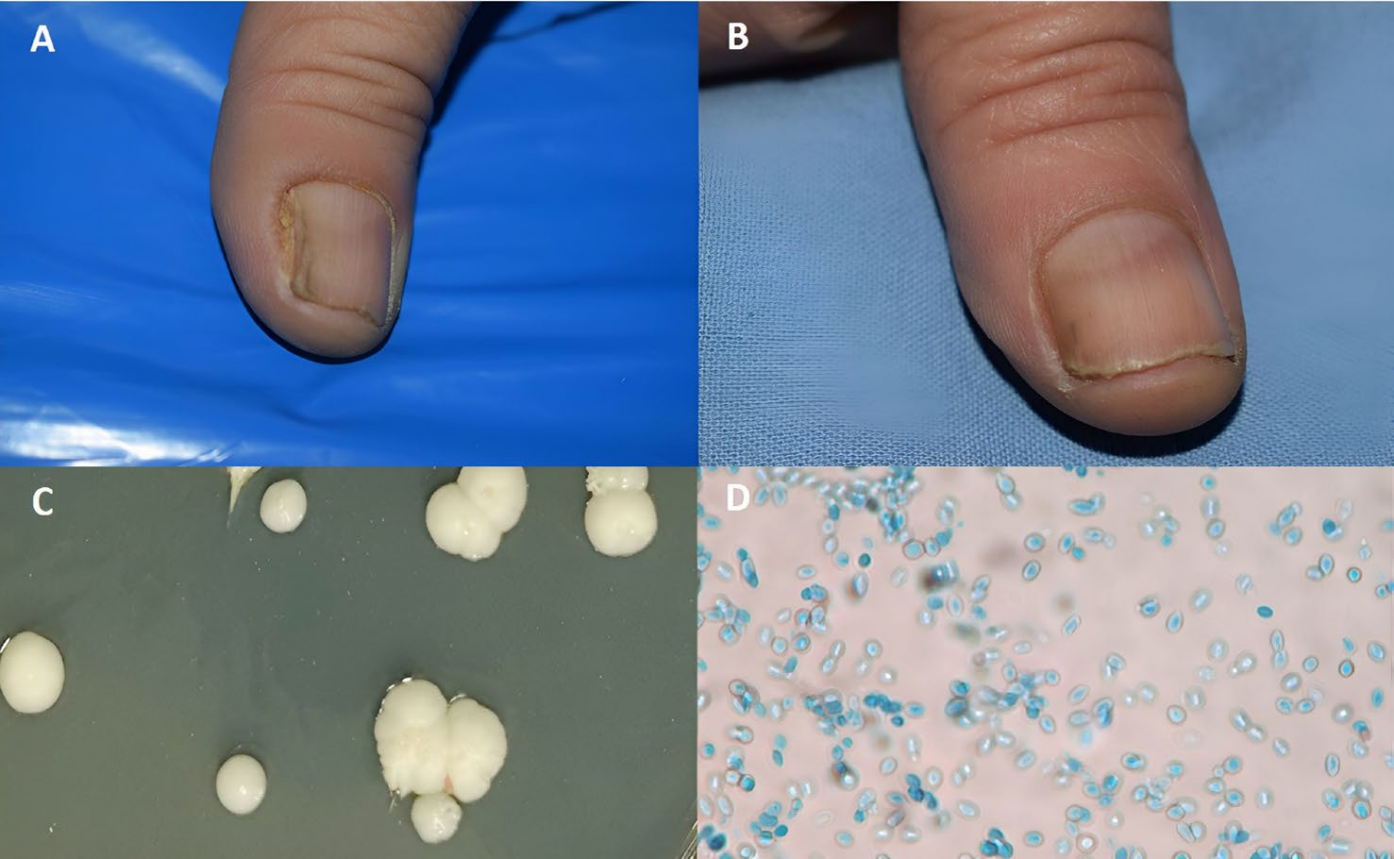
Patient with DLSO (A) Before treatment (B) After treatment with PDT treatment only (C) Culture on SDA without cycloheximide showing cream‐colored, raised, waxy colonies characteristic of yeast (D) Microscopic morphology of the colonies showing oval, cylindrical to ellipsoidal budding cells characteristic of yeast species (LCB mount ×400).

**FIGURE 3:**
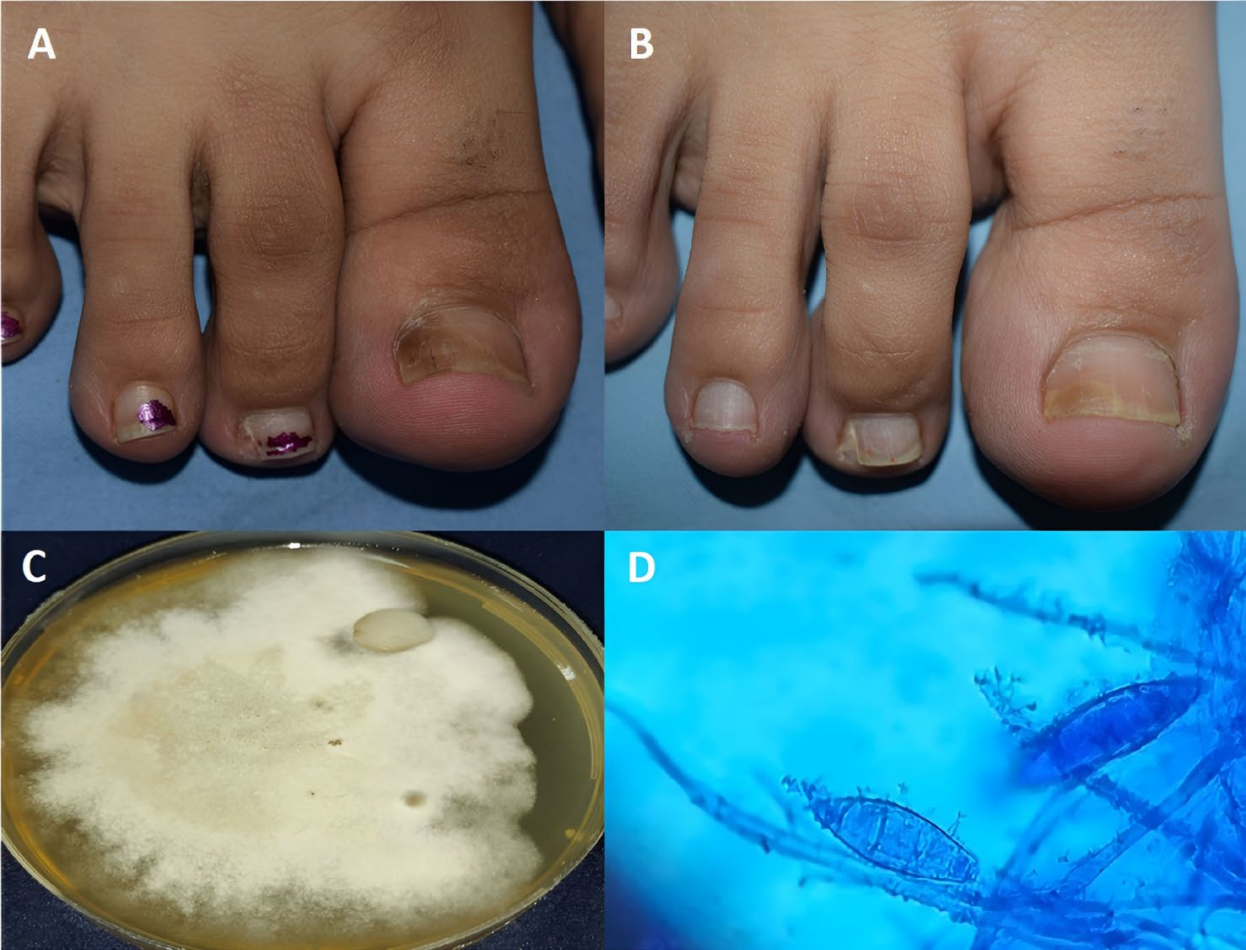
Patient with DLSO (A) Before treatment (B) After treatment with combined treatment (C) Culture on SDA without cycloheximide showing white to a yellowish colony with a coarsely fluffy, velvety texture (D) Microscopic morphology of the colonies showing spindle‐shaped macroconidia, with an asymmetrical apical knob consisted with *M. canis* (LCB mount ×400).

Fungal identification was based on growth rate, colony morphology (color, size, texture, and topography), and microscopic structures, including the type, size, shape, and arrangement of spores, as well as the size and color of hyphae (Figures 1, 2, 3).

The sample size was calculated using G* power software version (3.1.9.2). (A priori: Compute required sample size—given *α*, power and effect size), input parameters (effect size = 0.50), *α* error = 0.05, power (1−*β*) = 0.80 and Allocation ratio N2/N1 = 1 resulting in a sample size of 41; thus, the sample size increased to 45, 15 in each group. Statistical analyses were carried out using Statistical Program for Social Science (SPSS) version 24. The significance of the obtained results was judged at the *p* > 0.05 level.

### 2.1 Ethical Committee Registration Number

Der_70Med.Research_Photodynamic Therapy. Vs Its Combination Fractional Carbon Dioxide Laser/TTT.Onychomycosis_00000070.

## 3 Results

### 3.1 Demographic and Baseline Clinical and Mycological Characteristics

In this cohort, Group A (PDT) had a mean age of 41.6 years (±14.5), Group B (Fractional CO_2_) had a mean age of 36 years (±13.5), and Group C (Combined Treatment) had a mean age of 39.3 years (±9.5), with the study population overall averaging 39 years. Gender distribution skewed predominantly female, with 82% female and 18% male, and Group C showing a slightly higher proportion of males (33.3%) than Groups A and B (13.3% each).

Risk factors showed no significant variation among groups (*p* = 0.651). Prolonged nail moisture was reported in 53.3% of patients in Groups A and B, versus 40% in Group C. Frequent trauma was noted by 40% in Group A and 46.7% in Groups B and C (Table 3). Chronic diseases were more prevalent in Groups A (6.7%) and C (13.3%). Clinical onychomycosis types were diverse: DLSO (44.4%), TDO (28.9%), EO (13.3%), PSO (6.7%), mixed pattern (4%), and SWO (2.2%).

Mean disease duration differed significantly across groups (*p* = 0.03), with Group A at 1.86 ± 0.74 years, Group B at 2.26 ± 1.16 years, and Group C at 3.4 ± 2.5 years. In total, 58 fungal species were isolated, with no significant variation in fungal species distribution among groups. Yeast was the most common isolate (29.3%), followed by Aspergillus and *T. rubrum* (17.2% each), Penicillium (15.5%), and Trichosporon (10.3%). Less common isolates included *M. canis*, Rhodotorula, and *T. mentagrophytes* (3.4% each) (Table 2).

**TABLE 2:** Distribution of fungal species among the studied group.

Study group	PDT group	Fractional CO_2_ group	Combined group
**Fungal species**			
Aspergillus	3	2	5
*M. canis*	1	0	1
Penicillium	3	5	1
Rhodotorula	0	1	1
T. mentagrophytes	0	1	1
*T. rubrum*	5	1	4
Trichosporon	1	3	2
Yeast	7	4	6

*Note:* Some patients had more than one fungal isolate; thus, the total number of isolates exceeds the number of patients.

### 3.2 Follow‐Up Results (Tables 3, 4, 5)

**TABLE 3:** Follow‐up of onychomycosis severity index.

Severity index		Follow				χ^2^	*p*
		Before (*n* = 15)		After (*n* = 15)			
PDT group	Cure	0	0%	1	6.7%	6.86	0.076 NS
	Mild	1	6.7%	3	20%		
	Moderate	3	20%	7	46.7%		
	Severe	11	73.3%	4	26.7%		
Fractional CO_2_ group	Cure	0	0%	3	20%	7.77	0.051 NS
	Mild	2	13.3%	6	30%		
	Moderate	3	20%	2	13.3%		
	Severe	10	66.7%	4	26.7%		
Combined group	Cure	0	0%	3	20%	13.9	0.003 S
	Mild	3	20%	3	20%		
	Moderate	2	13.3%	8	53.3%		
	Severe	10	66.7%	1	6.7%		

*Note:* The reduction in onychomycosis severity index was significantly associated with the treatment modality (*p* = 0.003), with the combined group showing the most marked improvement. *P* vaue ≥ 0.05 : non significant (NS); *P* value ≤ 0.05 : significant (S).

**TABLE 4:** Follow‐up of KOH.

KOH		Follow				χ^2^	*p*
		Before (*n* = 15)		After (*n* = 15)			
PDT group	Negative	0	0%	9	60%	12.8	< 0.001S
	Positive	15	100%	6	40%		
Fractional CO_2_ group	Negative	0	0%	11	73.3%	17.4	< 0.001S
	Positive	15	100%	4	26.7%		
Combined group	Negative	0	0%	13	86.7%	22.9	< 0.001S
	Positive	15	100%	2	13.3		

*Note:* *P* vaue ≥ 0.05 : non significant (NS); *P* value ≤ 0.05 : significant (S).

**TABLE 5:** Follow‐up of fungal growth on SDA.

	Culture without cycloheximide					Culture with cycloheximide				
	Before ttt		After ttt		*p*	Before ttt		After ttt		*p*
	No.	%	No.	%		No.	%	No.	%	
PDT group	20	34.5	6	10.3	< 0.001	14	35.9	3	7.7	< 0.001S
Fractional CO_2_ group	17	29.3	4	6.9	< 0.001	10	25.6	2	5.1	0.004S
Combined group	21	36.2	2	3.4	< 0.001	15	38.5	2	5.1	< 0.001S

#### 3.2.1 Group A

The PDT group follow‐up showed a significant reduction in KOH positivity from 100% pretreatment to 40% posttreatment (*p* < 0.001), though changes in nail involvement extent were not statistically significant (*p* = 0.521). Posttreatment, 6.7% had no nail involvement, while 26.7% had 26%–50% and 76%–100% involvement. The proximity of infection to the nail matrix improved significantly (*p* = 0.008), with cases extending beyond three‐quarters of the distance to the matrix decreasing from 46.7% to 20%. While reductions in dermatophytoma presence and subungual hyperkeratosis were observed, these changes were not statistically significant (*p* = 0.543). Severity index analysis indicated an improvement, with severe cases dropping from 73.3% to 26.7%, though this trend did not reach statistical significance (*p* = 0.076).

#### 3.2.2 Group B

The Fractional CO_2_ group follow‐up showed a significant reduction in KOH positivity, dropping from 100% pretreatment to 26.7% posttreatment (*p* < 0.001), indicating effective fungal reduction. No statistically significant changes were observed in the extent of nail involvement (*p* = 0.105) or proximity of infection to the nail matrix (*p* = 0.062), suggesting a limited effect on infection spread. Dermatophytoma was initially present in one case, with no significant reduction posttreatment (*p* = 0.309). Subungual hyperkeratosis decreased from 60% to 26.7%, though not significantly (*p* = 0.065). Severity index trends indicated improvement, with some severe cases shifting to milder categories, but without statistical significance (*p* = 0.051).

#### 3.2.3 Group C

The combined treatment group showed a significant reduction in KOH positivity, from 100% to 13.3% posttreatment (*p* < 0.001). Nail involvement also improved notably (*p* = 0.004), with cases showing no involvement rising to 26.7%, while cases with 51%–75% involvement decreased from 46.7% to 13.3%. Infection proximity to the nail matrix improved significantly (*p* = 0.003). Minor, non‐significant reductions were observed in dermatophytoma (from 13.3% to 6.7%, *p* = 0.543) and subungual hyperkeratosis (from 26.7% to 13.3%, *p* = 0.361). The severity index showed significant improvement (*p* = 0.003), with severe cases decreasing from 66.7% to 6.7%, and increases in moderate and mild cases to 53.3% and 33.3%, respectively.

### 3.3 Patient Satisfaction

Posttreatment satisfaction levels showed no significant differences among groups (*p* = 0.198). In the PDT group, 46.7% were dissatisfied, 40% mildly satisfied, and 13.3% fully satisfied. For the Fractional CO_2_ group, 33.3% reported dissatisfaction, 26.7% mild satisfaction, and 40% full satisfaction. The combined treatment group had 13.3% dissatisfied, 40% mildly satisfied, and 46.7% fully satisfied.

### 3.4 Side Effects

The incidence of side effects was similar across groups (*p* = 0.649). Group A also had 66.7% with side effects, mainly discoloration. In Group B, 66.7% experienced side effects, with 60% reporting pain and 40% burn marks. In Group C, 80% reported side effects, including 50% with discoloration, 75% with pain, and 25% with burn marks. However, these side effects did not interfere with continuing the treatment.

## 4 Discussion

Onychomycosis is famed for being difficult‐to‐treat infection because of the deep‐seated nature of the fungus within the nail plate, the prolonged treatment required for resolution, poor patient compliance, and frequent recurrence [1].

Expanding treatment options are required to overcome the lack of response and reduce the relapse and adverse effects of conventional systemic antifungals. Therapeutic options based on devices such as lasers, intense pulsed light (IPL), iontophoresis, and PDT could help to prevail over the limitations mentioned above [8].

This study aimed to assess the safety and effectiveness of photodynamic therapy (PDT) alone, fractional CO_2_ laser alone, and the combination of both for treating onychomycosis.

Despite being more commonly prevalent in the middle age group 41–60 years [9]. Our study showed that onychomycosis was more diagnosed in the age group between 21 and 40 years with the overall mean age of the patients 39 years, consistent with Jha et al. where the most prevalent age group was between 21 and 60years [10].

In contrast to previous studies which showed male patients' predominance [11], our study had an exceeding number of females (82%). A recently published Iranian epidemiological study has revealed that more females are affected than males [12]. Lipner et al. [13] explained in their recent study that females usually seek more medical advice than males. Also, from an esthetic point of view, females are more often concerned with unacceptable cosmetic changes than males.

Although most studies on onychomycosis showed that toenail affection is more common than fingernail affection [14, 15], we have different findings; 71% of our patients have fingernail affection. The reason could be the increased number of female patients who are more often exposed to moist and household work, and known to have more fingernails than toenails onychomycosis.

In the same context, prolonged moisture was the most prevalent risk factor in this study; this finding is consistent with the number of female patients and the most affected nails. However, this result is not on the same track as previous research that studied the risk factors for onychomycosis and found that aging and poor peripheral circulation are the most common factors [16]. We explain that the common age in our research was females in the child‐bearing period, whose exposure to moisture is more often than in other age groups.

We also found that the DLSO type is the most common presentation, with a percentage of 40%, which is consistent with a previous Indian study [17]. TDO comes in second place at 28% of the total number of patients, which may indicate chronicity of the disease in some patients.

Most studies revealed that onychomycosis is caused mainly by dermatophytes in up to 75% of fingernails and 90% of toenails [1]. However, the present study found that yeast species are the most common etiological agents, consistent with previous Egyptian studies [18, 19]. These results may indicate that yeast species may be more common etiological agents in warmer climates.

When assessing treatment efficiency for each group, we found that PDT alone leads to mycological cure in 60% of patients, but a complete cure was seen only in one patient, which is different from Alberdi et al. (2019) whose complete cure was 70% after 12 weeks of treatment [20]. Their pretreatment use of urea 40% for a longer period (3–7 days) may play a role in a higher mycological and clinical cure rate, as urea, in concentrations over approximately 30%, is recognized as a keratolytic agent that softens and hydrates the nail plate by breaking down nail keratin, thereby improving drug penetration and facilitating the removal of affected nails [21].

On the other hand, the group treated with fractional CO_2_ alone showed only 20% complete cure, despite the mycological cure being 73.3%, which means that this modality may achieve a high enough temperature for killing fungal elements. However, a low percentage of clinical cures matches a recent Indian study that explained that higher energies may not be clinically tolerated and could damage the underlying nail bed [22].

Although the combination group showed a higher mycological cure (86.7%), the complete cure was only 20%. However, the improvement in the onychomycosis severity index was significant in contrast to the other two previous groups, where the improvement was non‐significant, which explains the higher percentage of satisfaction among this group of patients. A recent Egyptian study about toenail onychomycosis concluded different results; they found a non‐significant difference between PDT alone and Fractional CO_2_‐assisted PDT when comparing the two modalities in the same individual. However, the improvement in the severity index in their study was significant in both groups [23]. It may indicate that risk factors for onychomycosis in each patient may play a role in the response to a certain treatment modality and poor response in other individuals.

To our knowledge, the only study that compares the three different modalities found that combining fractional CO_2_ laser with PDT significantly improved clinical outcomes in patients with onychomycosis compared to either therapy alone [24]. This supports our findings and suggests that the combined therapy's synergistic effect enhances treatment efficacy.

## 5 Limitations

Despite the compelling evidence supporting combined therapy, it is important to acknowledge the study's limitations. The sample size was small, and the study duration may not have captured long‐term outcomes. Additionally, the study did not explore the combined therapy's cost‐effectiveness, an important consideration for broader clinical adoption.

## 6 Conclusion

In conclusion, combining PDT and fractional CO_2_‐assisted drug delivery offers an effective and safe treatment option for onychomycosis, surpassing the efficacy of either treatment alone. This approach provides significant mycological cure, clinical improvements, and higher patient satisfaction, although a longer course of treatment may be needed to achieve a complete cure. Incorporating combined therapy into clinical practice could enhance treatment outcomes for patients with onychomycosis.

## Author Contributions

This study was a collaborative effort among four authors. Dr. Hamed Abdou conceptualized the study, supervised the research process, and contributed significantly to data analysis. Dr. Shady Mahmoud Ibrahim was the primary investigator, overseeing clinical procedures, data interpretation, and manuscript preparation. Dr. Mostafa Taha Eldestawy managed patient recruitment, data collection, and statistical evaluation while contributing to the literature review. Dr. Muhammad Mohsen assisted with data acquisition, performed laboratory analyses, and contributed to drafting and revising the manuscript. All authors critically reviewed and approved the final manuscript for publication.

## Consent

All participants gave informed consent before participating in the study. Documentation of consent is available upon request.

## Conflicts of Interest

The authors declare no conflicts of interest.

## Data Availability

Data supporting this study's findings are available from the corresponding author upon reasonable request, ensuring compliance with relevant ethical guidelines.

## References

[1] A. K. C. Leung , J. M. Lam , K. F. Leong , et al., “Onychomycosis: An Updated Review,” Recent Patents on Inflammation & Allergy Drug Discovery 14, no. 1 (2020): 32–45. 31738146 10.2174/1872213X13666191026090713 PMC7509699

[2] S. R. Lipner and R. K. Scher , “Onychomycosis: Clinical Overview and Diagnosis,” Journal of the American Academy of Dermatology 80, no. 4 (2019): 835–851. 29959961 10.1016/j.jaad.2018.03.062

[3] A. K. Gupta , R. R. Mays , S. G. Versteeg , N. H. Shear , and V. Piguet , “Update on Current Approaches to Diagnosis and Treatment of Onychomycosis,” Expert Review of Anti‐Infective Therapy 16, no. 12 (2018): 929–938. 30411650 10.1080/14787210.2018.1544891

[4] J. J. Shen , G. B. E. Jemec , M. C. Arendrup , and D. M. L. Saunte , “Photodynamic Therapy Treatment of Superficial Fungal Infections: A Systematic Review,” Photodiagnosis and Photodynamic Therapy 31 (2020): 101774. 32339671 10.1016/j.pdpdt.2020.101774

[5] W. H. S. Ng and S. D. Smith , “Laser‐Assisted Drug Delivery: A Systematic Review of Safety and Adverse Events,” Pharmaceutics 14, no. 12 (2022): 2738. 36559233 10.3390/pharmaceutics14122738 PMC9787022

[6] A. Warrier , N. Mazumder , S. Prabhu , K. Satyamoorthy , and T. S. Murali , “Photodynamic Therapy to Control Microbial Biofilms,” Photodiagnosis and Photodynamic Therapy 33 (2021): 102090. 33157331 10.1016/j.pdpdt.2020.102090

[7] C. Carney , A. Tosti , R. Daniel , et al., “A New Classification System for Grading the Severity of Onychomycosis: Onychomycosis Severity Index,” Archives of Dermatology 147, no. 11 (2011): 1277–1282. 22106113 10.1001/archdermatol.2011.267

[8] P. Robres , C. Aspiroz , A. Rezusta , and Y. Gilaberte , “Usefulness of Photodynamic Therapy in the Management of Onychomycosis,” Actas Dermo‐Sifiliográficas 106, no. 10 (2015): 795–805. 26427737 10.1016/j.ad.2015.08.005

[9] M. Papini , B. M. Piraccini , E. Difonzo , and A. Brunoro , “Epidemiology of Onychomycosis in Italy: Prevalence Data and Risk Factor Identification,” Mycoses 58, no. 11 (2015): 659–664. 26412300 10.1111/myc.12396

[10] B. Jha , M. Sharma , S. Gc , and J. Sapkota , “Onychomycosis Among Clinically Suspected Cases Attending the Dermatology out‐Patient Department of a Tertiary Care Centre: A Descriptive Cross‐Sectional Study,” JNMA: Journal of the Nepal Medical Association 59, no. 237 (2021): 450–453. 34508426 10.31729/jnma.6277 PMC8673452

[11] S. Gregoriou , N. Mpali , G. Vrioni , E. Hatzidimitriou , S. E. Chryssou , and D. Rigopoulos , “Epidemiology of Onychomycosis in an Academic Nail Unit in South Greece During a Three‐Year Period,” Skin Appendage Disorders 6, no. 2 (2020): 102–107. 32258053 10.1159/000504812 PMC7109406

[12] T. Razavyoon , S. J. Hashemi , P. Mansouri , et al., “The Epidemiology and Etiology of Onychomycosis in 2 Laboratory Centers Affiliated to Tehran University of Medical Sciences During 2019–2020,” Iran Journal of Microbiology 14, no. 2 (2022): 268–275. 10.18502/ijm.v14i2.9196 PMC9168250 35765553

[13] S. R. Lipner , J. M. Falotico , and A. Konda , “On the Basis of Sex: Impact and Treatment of Toenail Onychomycosis in Female Patients,” Journal of Clinical and Aesthetic Dermatology 16, no. 10 (2023): 52–57. PMC10617899 37915332

[14] S. Nikitha , N. Kondraganti , and V. Kandi , “Total Dystrophic Onychomycosis of All the Nails Caused by Non‐Dermatophyte Fungal Species: A Case Report,” Cureus 14, no. 9 (2022): e29765. 36324367 10.7759/cureus.29765 PMC9618008

[15] P. Halteh , R. K. Scher , and S. R. Lipner , “Over‐The‐Counter and Natural Remedies for Onychomycosis: Do They Really Work?,” Cutis 98 (2016): e16–e25. 28040821

[16] B. E. Elewski and A. Tosti , “Risk Factors and Comorbidities for Onychomycosis: Implications for Treatment With Topical Therapy,” Journal of Clinical and Aesthetic Dermatology 8, no. 11 (2015): 38–42. PMC4689496 26705439

[17] P. Yadav , A. Singal , D. Pandhi , and S. Das , “Clinico‐Mycological Study of Dermatophyte Toenail Onychomycosis in New Delhi, India,” Indian Journal of Dermatology 60, no. 2 (2015): 153–158. 25814703 10.4103/0019-5154.152511 PMC4372907

[18] E. B. Abd El‐Aal , H. M. Abdo , S. M. Ibrahim , et al., “Fractional Carbon Dioxide Laser Assisted Delivery of Topical Tazarotene Versus Topical Tioconazole in the Treatment of Onychomycosis,” Journal of Dermatological Treatment 30, no. 3 (2019): 277–282. 30081698 10.1080/09546634.2018.1509046

[19] A. M. Zaki , H. M. Abdo , M. A. Ebadah , and S. M. Ibrahim , “Fractional CO_2_ Laser Plus Topical Antifungal Versus Fractional CO_2_ Laser Versus Topical Antifungal in the Treatment of Onychomycosis,” Dermatologic Therapy 33, no. 1 (2020): e13155. 31697010 10.1111/dth.13155

[20] E. Alberdi and C. Gómez , “Efficiency of Methylene Blue‐Mediated Photodynamic Therapy vs Intense Pulsed Light in the Treatment of Onychomycosis in the Toenails,” Photodermatology, Photoimmunology & Photomedicine 35, no. 2 (2019): 69–77. 10.1111/phpp.12420 30168611

[21] S. Dars , H. A. Banwell , and L. Matricciani , “The Use of Urea for the Treatment of Onychomycosis: A Systematic Review,” Journal of Foot and Ankle Research 12 (2019): 22. 31007722 10.1186/s13047-019-0332-3 PMC6458736

[22] C. Grover , S. Nanda , and S. Bansal , “Efficacy of Fractional CO_2_ Laser in Onychomycosis: A Clinical Evaluation,” Indian Dermatology Online Journal 13, no. 1 (2021): 133–134. 35198487 10.4103/idoj.IDOJ_77_21 PMC8809178

[23] M. Abdallah , M. M. Abu‐Ghali , M. T. El‐Sayed , et al., “Fractional CO_2_‐Assisted Photodynamic Therapy Improves the Clinical Outcome and Patient's Satisfaction in Toenail Onychomycosis Treatment: An Intra‐Patient Comparative Single‐Center Study,” Journal of Dermatological Treatment 33, no. 1 (2022): 542–549. 32419540 10.1080/09546634.2020.1771252

[24] N. Sobhy , H. Talla Eweed , and S. S. Omar , “Fractional CO_2_ Laser ‐ Assisted Methylene Blue Photodynamic Therapy Is a Potential Alternative Therapy for Onychomycosis in the Era of Antifungal Resistance,” Photodiagnosis and Photodynamic Therapy 40 (2022): 103149. 36228978 10.1016/j.pdpdt.2022.103149

